# Effect of C1q/TNF-Related Protein 9 on Coronary Artery Calcification: An Observational Study

**DOI:** 10.3390/jcdd9100313

**Published:** 2022-09-20

**Authors:** Demin Liu, Yanan Ma, Xiaoxue Jin, Rui Lu, Haijuan Hu, Guoqiang Gu

**Affiliations:** Department of Cardiology, The Second Hospital of Hebei Medical University, Heping West Road No. 215, Shijiazhuang 050000, China

**Keywords:** CTRP9, type 2 diabetes mellitus, coronary artery calcification, intravascular optical coherence

## Abstract

Coronary artery calcification (CAC) increases the risk of acute coronary syndrome. This study examined the correlation between C1q/TNF-related protein 9 (CTRP9) and CAC and explored CTRP9 as a biomarker for prognosis. We divided 275 patients with coronary heart disease into four groups. In order to balance the baseline confounding factors, propensity score matching (PSM) was performed to match CAC patients with non-CAC patients in a 1:1 ratio. Optical coherence tomography (OCT) calcification scoring was performed in 126 patients with CAC. Moreover, 140 patients who underwent OCT were followed-up for 9 months for analysis of the correlation between CTRP9 levels and clinical prognosis. Based on OCT calcification scores, 126 patients with CAC were divided into the 0–2 and 3–4 groups. Plasma CTRP9 levels were significantly lower in the type 2 diabetes mellitus (T2DM), CAC and CAC with T2DM groups than in the control group. CTRP9 played roles as a protective factor and potential predictor in CAC severity. The AUC of the OCT calcification score 3–4 group predicted by the plasma CTRP9 level was 0.766. During the follow-up period, the cumulative event-free survival rate was significantly lower in the low-level CTRP9 (L-CTRP9) group than in the high-level (H-CTRP9) group, and the incidence of major endpoint events was significantly higher in the L-CTRP9 group than in the H-CTRP9 group. CTRP9 can be a valuable biomarker for CAC occurrence and severity and can predict patients’ clinical prognosis.

## 1. Introduction

Vascular calcification is a common pathophysiological process in atherosclerosis, type 2 diabetes mellitus (T2DM), aging, and chronic kidney disease [1]. Epidemiological investigations show that coronary artery calcification (CAC) increases with age, with an incidence of approximately 50% and 80% in individuals aged 40–49 and 60–69 years, respectively [2]. Researchers have gradually realized that calcification is an active inflammatory process regulated by multiple signaling pathways with calcification evaluation and diagnostic technology development. Vascular calcification can occur in different vascular arterial layers. Intimal calcification usually coexists with atherosclerotic plaques [3]. Therefore, intimal calcification can affect coronary atherosclerotic plaque stability and is the main risk factor for interventional surgery failures and complications. Calcification of the middle arterial layer is mostly associated with metabolism-related diseases [4,5]. Coronary artery media calcification can reduce the degree of the coronary artery relaxation and contraction response to physiological stimulation and affect coronary blood-flow regulation, leading to adverse cardiovascular events. Calcification usually formed in patients with T2DM is characterized by the coexistence of intimal and media calcifications [6]. Moreover, studies have shown that CAC can not only increase the risk of interventional cardiology, but also increase the risk of stroke and ischemic heart disease, but it is also the leading cause of death, cardiac death, and valvular calcification [7,8].

Moreover, related studies have shown that compared to non-diabetic patients, patients with T2DM have a significantly higher risk of developing CAC. In addition, the proportion of moderate and severe calcification in patients with T2DM is also significantly higher [9]. CAC is a pathological process and an all-cause mortality predictor in patients with diabetes [3]. Therefore, more attention should be given to patients with T2DM and CAC. Current research on the CAC mechanism has gone deep from morphology to molecular biology; therefore, identifying the corresponding molecular markers has a very important clinical value for the early diagnosis, prevention, and intervention of CAC.

C1q/TNF-related protein 9 (CTRP9) is a newly discovered adipose factor highly homologous to adiponectin and highly expressed in adult hearts [10]. Many studies have shown that CTRP9 plays a beneficial role in cardiovascular disease by regulating glucose and lipid metabolism and vasodilation; inhibiting inflammation, oxidative stress, and vascular aging; protecting endothelial cells; delaying atherosclerosis occurrence; and reducing myocardial ischemia-reperfusion injury [11,12,13,14]. However, the role of CTRP9 expression in the development and progression of CAC is still unclear. In this study, we explored the relationship between CTRP9 and CAC and evaluated its potential as a clinical prognostic indicator for patients with CAC in order to investigate new biological indicators for early CAC identification and diagnosis.

Currently, the methods and techniques used to diagnose and evaluate CAC include coronary angiography (CAG), computed tomography (CT), intravascular optical coherence tomography (OCT), and intravascular ultrasound (IVUS) [15]. CT has a high sensitivity for identifying calcification and could well evaluate the overall calcification load, but there are limitations for the detection of punctate calcification, and CT cannot accurately evaluate the morphological characteristics of CAC in the lumen. Most importantly, for patients with tachycardia and arrhythmia, the quality of coronary CT imaging is poor, not only that, this test also has more contraindications, such as pregnant women and patients who cannot cooperate with scanning and holding their breath. Intracavitary imaging technology has attracted much attention for plaque recognition and preoperative evaluation because of its high resolution and contrast. Compared with CT, OCT could distinguish intimal calcification from adventitia calcification. Not only that, it can also penetrate calcium ions, measure calcium thickness, and evaluate the range of three-dimensional calcification [16]. Considering this, Fujino et al. developed a calcification scoring system based on OCT to evaluate calcification severity, optimize the CAC treatment strategy, and predict stent expansion [17,18]. The poor stent expansion is an important risk factor for increasing in-stent thrombosis, in-stent restenosis, and major adverse cardiovascular events (MACE) [19,20]. In summary, the OCT calcification score can be used to distinguish calcification severity and evaluate the clinical prognosis of patients with calcification. Because OCT is an invasive and expensive method, it is of great clinical value to explore the correlation between CTRP9 and CAC severity and its effect on prognosis.

## 2. Materials and Methods

### 2.1. Participants and Eligibility

**Figure 1:**
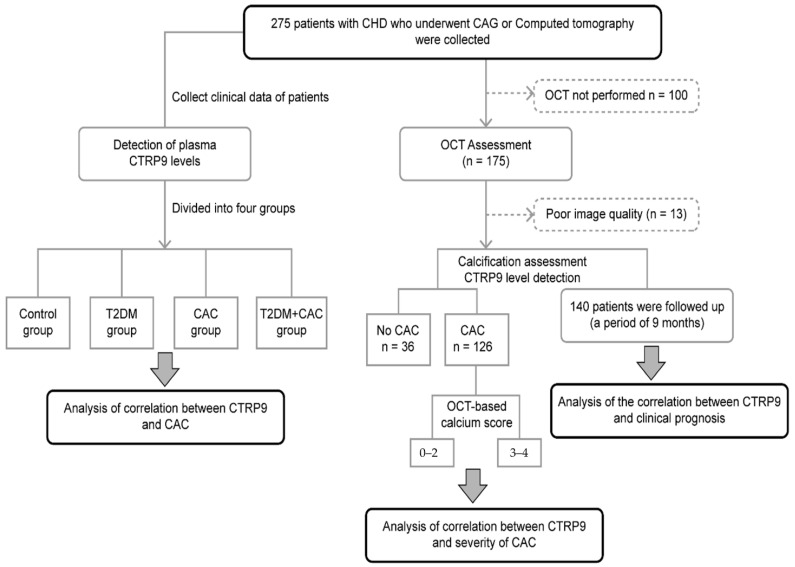
Flow chart to study the relationship between CTRP9 and coronary artery calcification.

In this observational study, from December 2020 to April 2021, we enrolled 275 patients with coronary heart disease consulting at the Department of Cardiology, Second Hospital of Hebei Medical University. CAC was diagnosed using CAG or CT. The patients were divided into four groups according to T2DM and CAC presence: the control (n = 59), T2DM (n = 36), CAC (n = 92), and CAC with T2DM groups (n = 88). Propensity score matching (PSM) was performed to match CAC patients with non-CAC patients in a 1:1 ratio (non-CAC (n = 85) and CAC groups (n = 180)). According to the OCT calcification score, patients with CAC who underwent OCT examination were divided into the 0–2 and 3–4 subgroups. According to the CTRP9 cutoff value, patients undergoing OCT examination and follow-up were divided into the high- and low-level CTRP9 groups. This research protocol was according to the ethical code of the Declaration of Helsinki. This study was reviewed and approved by the Second Hospital of Hebei Medical University Ethics Committee. Patients with the following diseases were excluded: (1) type 1 diabetes and other special types of diabetes; (2) myocarditis, severe arrhythmias, and other heart diseases; (3) severe infectious diseases and major organ dysfunction; and (4) autoimmune diseases and malignant tumors (Figure 1).

### 2.2. Clinical and Biochemical Characteristics of the Study Population

General clinical data included sex, age, hypertension history, body mass index (BMI), heart rate (HR), blood pressure (BP), left ventricular ejection fraction (LVEF), *E*/*A* ratio, and end-diastolic volume (EDV). Laboratory examination data included glycosylated hemoglobin (HbA1c), fasting blood glucose (GLU), total cholesterol (TC), serum triglycerides (TG), high-density lipoprotein (HDL-C), low-density lipoprotein (LDL-C), creatinine (SCR), myocardial enzymes, creatinine kinase (CK), creatine kinase-muscle/brain (CK-MB), γ-glutamyl transpeptidase (GGT), hypersensitive C-reactive protein (hsCRP), HDL-C, TC, TG, LDL-C, alanine transaminase (ALT), apolipoprotein B (ApoB), lipoprotein a (LPa), hemoglobin (HGB), end-systolic volume (ESV), apolipoprotein A1 (APOA), lactate dehydrogenase (LDH), myoglobin (MYO), SCR, β2 microglobulin (β2MG), the ratio of transmitral early peak velocity to e’ (E/e’), and CTRP9.

### 2.3. Detection of Plasma CTRP9 Level

The patient’s plasma CTRP9 level was determined using ELISA (Cat, EIA1322H for CTRP9, Elisa Biotech, Shanghai, China), strictly according to the manufacturer’s instructions.

### 2.4. Optical Coherence Tomography Image Acquisition and Analysis

#### 2.4.1. OCT Image Acquisition

After the intracoronary nitroglycerin injection, OCT images were obtained using a frequency domain OCT system (Abbott Vascular, Santa Clara, CA, USA). The traditional angioplasty guide wire (0.014 in) was pushed to the distal end of the area of interest, and then the OCT imaging catheter (Dragonfly, Abbott Vascular, Santa Clara, CA, USA) was pushed beyond the area of interest on the guide wire. Approximately 10–14 mL of contrast medium was needed to remove blood cells from the target vessels during image acquisition. Generally, in patients with myocardial infarction thrombolysis (TIMI) grades 2 and 3, OCT is performed before any interventional therapy, and for patients with TIMI flow grade 0 or 1, thrombus aspiration is performed before OCT imaging. The image was calibrated by automatically adjusting the Z offset and automatically pulling it back to 20 mm/sly. The angiographic features that led to the study exclusion were left trunk involvement >50%, lesions with severe calcification, culprit lesions in bypass vessels, obstructions to OCT catheter passage, severely tortuous vessels, and culprit lesions located very far away [21,22,23].

#### 2.4.2. OCT Image Analysis, including Calcium Parameters

We used a special software (Off-line Review Software, version E.0.2, Abbott Vascular, Santa Clara, CA, USA) for OCT image analysis. All cross-sectional images were initially screened by quality assessment, including lateral branches occupying more than 30° of the cross-section or poor quality due to residual blood, artifacts, or reverberation, which needed to be excluded from the analysis.

**Figure 2:**
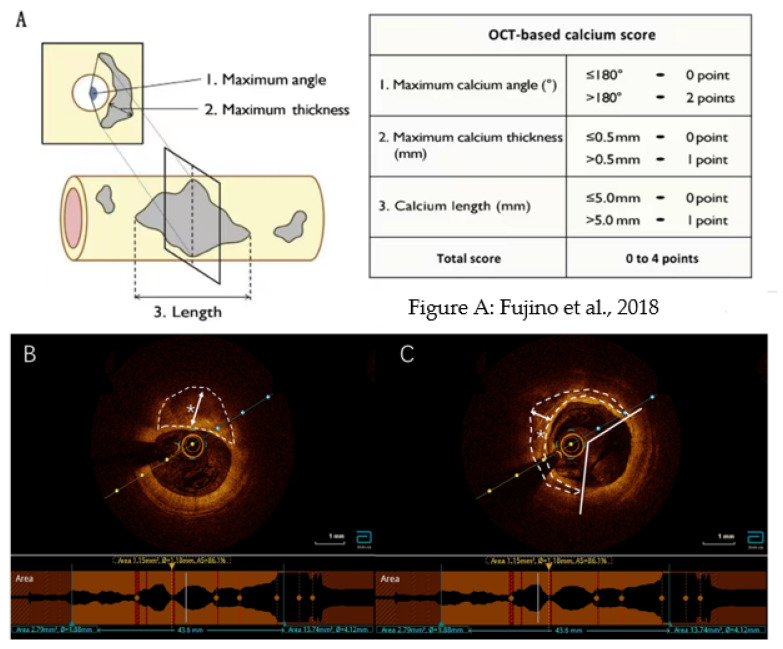
Calcification score based on optical coherence tomography. (**A**) Calcification scoring system based on optical coherence tomography (OCT) [17]. The OCT-based calcium score consists of three parameters obtained from the OCT images: maximum calcium angle, maximum calcium thickness, and calcium length. Each parameter was assigned 1 or 2 points, and the total score (0–4) was calculated. (**B**,**C**) Typical OCT calcification images. Calcium (*): The distance between the inner edge of calcium and the lumen surface is the calcification thickness, as shown by the white arrow, and the calcification angle is measured by a protractor centered on the lumen, as shown by a white straight line. OCT: optical coherence tomography.

Quantitative calcification indicators were evaluated and measured at intervals of 1 mm in each OCT frame as shown in Figure 2. First, we measured the calcium angle and maximum calcium thickness in each cross-section. Then, we chose the maximum calcium angle cross-section as the representative target calcium cross-section and measured the calcium angle, maximum calcium thickness, and longitudinal calcium length in the cross-section (Figure 2).

On this basis, Fujino et al. developed a calcification scoring system based on OCT that aimed to evaluate calcification severity and predict the stent expansion effect. Fujino et al. used three parameters (maximum angles, maximum thickness, and length) to evaluate each calcium deposition type in the target lesion using OCT. Fujino et al. assigned a score of 1 to 2 for each of the following conditions: maximum calcification angle of > 180° = 2, maximum calcification thickness > 0.50 mm = 1, and calcification length > 5 mm = 1. The total score (0–4) was calculated (Figure 2). On this basis, subjects with CAC shown by OCT were divided into two groups according to CAC severity: the 0–2 and 3–4 group. To ensure the accuracy of the experiment, two groups of patients were retested for CTRP9.

All OCT data were analyzed using dedicated offline review systems (Abbott Vascular or Terumo) and expert consensus reports by three independent interventional cardiologists and reviewed by a third reader blinded to the clinical and angiographic information.

### 2.5. Follow-Up

Trained doctors followed up with the discharged subjects regularly for 1, 3, 6 and 9 months, and then annually after that. Patients were admitted to the hospital for professional evaluation and treatment if adverse events occurred during follow-up. Endpoints and definitions: The main end-events of follow-up were cardiogenic death, unstable angina pectoris, myocardial infarction, or hospitalization with PCI and coronary artery bypass grafting. The first-occurrence primary end-event was defined as event-free survival time and the secondary end-event as rehospitalization for heart failure.

Health-status assessment: At 9 months, the participant’s health status was investigated using the Seattle Angina Questionnaire (SAQ). The SAQ is a 19-item questionnaire used to assess symptoms, function, and quality of life in patients with coronary heart disease and comprises five parts: physical limitation, angina stability, angina frequency, QoL, and treatment satisfaction [24]. The higher the score in each field, the lower the symptom burden, the better the physical function, and the better the QoL. Since patients with diabetes may have atypical angina symptoms, we also examined the association between diabetes and dyspnea symptoms using the Rose Dyspnea Core (RDS) score. The RDS is a four-item survey that assesses dyspnea caused by common activities [25]. The higher the score, the greater the dyspnea restrictions.

### 2.6. Statistical Analysis

Continuous variables conforming to a normal distribution are presented as the mean ± SD and were compared among the four groups using factorial analysis of variance (ANOVA) with the least significant difference (LSD) test for multiple pairwise comparisons. Median and quartile spacing (M (QL, QU)) was used for non-normal distribution measurement data, and the rank-sum test was used to compare groups. Categorical variables were expressed as number of cases and percentages and were compared between groups using the chi-square or Fisher’s exact test (expected cell value <5). Multivariate regression analysis was used to describe the relationship between risk factors and CAC. The receiver operating characteristic (ROC) curve and area under the ROC curve (AUC) were used to evaluate CTRP9 and CAC predictive value and CAC severity. The Kaplan–Meier method was used as a time-event variable to construct survival curves to evaluate patient prognosis. Statistical analyses were performed using SPSS 26.0 (IBM Corp., Armonk, NY, USA). Statistical significance was set at *p* < 0.050.

To balance the baseline confounding factors, we performed propensity score (PS) matching analysis using the nearest-neighbor matching algorithm with a ratio of 1:1 and calipers of width equal to 20% SDs of the estimated PS after logit transformation. The PS estimation was identified by a non-parsimonious multivariable logistic regression using variables that were determined by clinical experience and univariate Cox regression analyses including sex, age, hypertension history, diabetes, and LDH.

## 3. Results

### 3.1. Baseline Characteristics

The general clinical data of the participants are summarized in Table 1. This study included 275 patients. No significant differences were noted in the CK, CK-MB, GGT, hsCRP, HDL-C, TC, TG, LDL-C, ALT, ApoB, LPa, HGB, LVEF, *E*/*A* ratio, EDV or ESV of the patients among the four groups. Significant differences were noted in sex, age, smoking history, hypertension history, heart failure, HbA1c, GLU, BMI, HR, AST, APOA, LDH, MYO, SCR, β2 microglobulin (β2MG), E/e’ and CTRP9 (*p* < 0.050) among the four groups. The plasma CTRP9 levels in the CAC, T2DM and CAC with T2DM groups were significantly lower than those in the control group (*p* < 0.001). In addition, the plasma CTRP9 level was significantly lower in patients with CAC and T2DM than in patients in the CAC group (68.1 vs. 80.9, *p* < 0.001).

### 3.2. CTRP9 Is a Protective Factor for CAC

In order to remove the interference of confounding factors, propensity score matching (PSM) was performed to match CAC patients with non-CAC patients in a 1:1 ratio. In baseline characteristics, 95 participants from the non-CAC group were matched at a 1:1 ratio to 180 participants from the CAC group. After PSM, compared with non-CAC group, the CAC group had lower CTRP9 levels, *p* < 0.05.

### 3.3. Plasma CTRP9 Diagnostic Value for CAC

**Figure 3:**
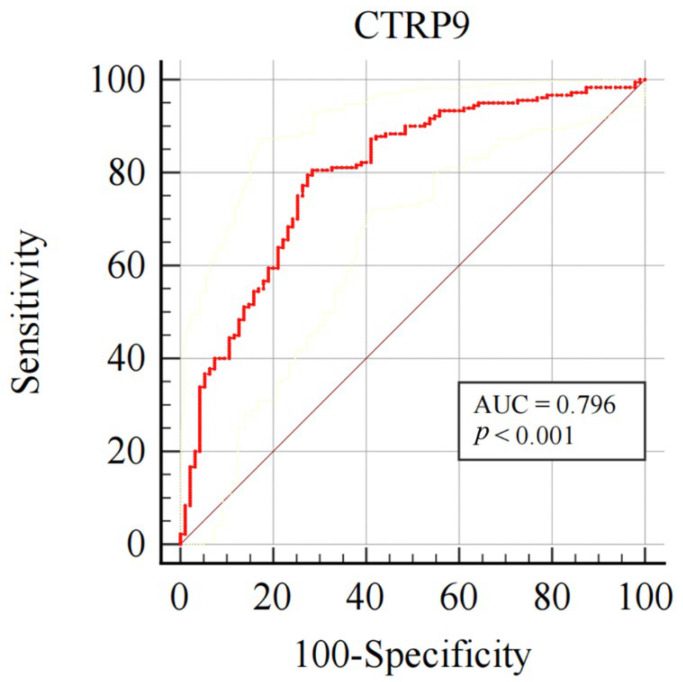
Predictive value of plasma CTRP9 level on CAC. Receiver operating characteristic (ROC) curves for predicting CAC. The AUC was 0.796 (95% CI: 0.744–0.842, *p* < 0.001), the cut-off value was 109.17 pg/mL (sensitivity 80.6%, specificity 71.6%). CI, confidence interval; AUC, area under the curve. CTRP9 and C1q/TNF-related protein 9 expression.

ROC curve analysis showed that plasma CTRP9 levels could be used to predict CAC presence. The AUC of the ROC curve was 0.796 (95% CI 0.744–0.842, *p* < 0.001). The CTRP9 value of 109.17 pg/mL was the best threshold value for identifying CAC which had strong specificity (sensitivity 80.6%, specificity 71.6%) (Figure 3). After PS matching, similar results were obtained (Table 2).

### 3.4. Correlation Analysis between CTRP9 and Severity of Coronary Artery Calcification

**Figure 4:**
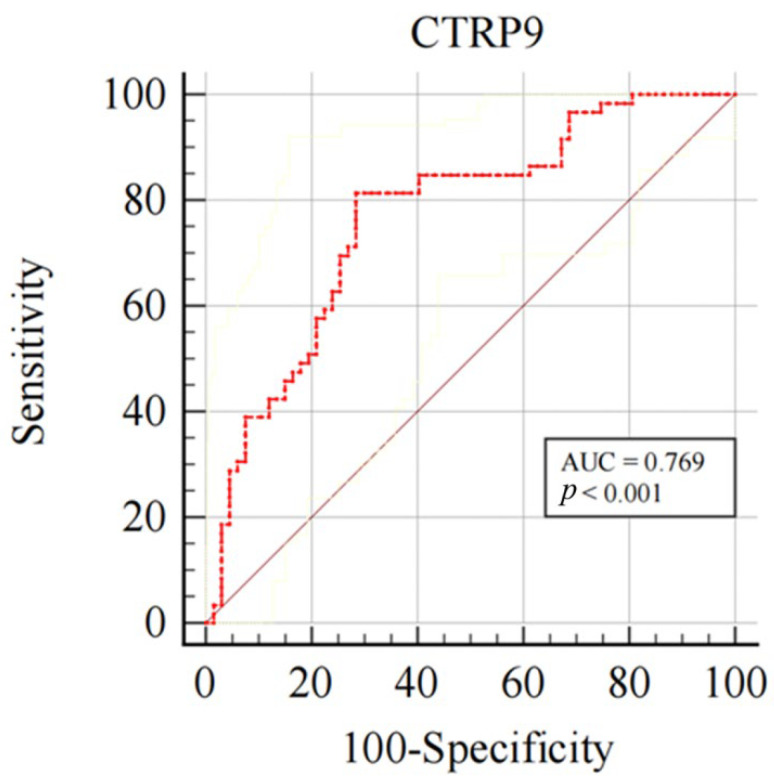
Predictive value of plasma CTRP9 level on calcification severity. Receiver operating characteristic (ROC) curves for predicting calcification severity. The AUC was 0.769 (95% CI: 0.685–0.839, *p* < 0.001), the cut-off value was 89.13 pg/mL (sensitivity 81.40%, specificity 71.60%). CI, confidence interval; CTRP9, C1q/TNF-related protein 9.

Of the 275 participants, 175 underwent OCT because of illness. The OCT image quality was poor in 13 cases and non-calcified in 36. Finally, 126 patients with calcified OCT images were analyzed. According to Fujino et al., the calcification score system based on OCT divides it into the 0–2 and 3–4 subgroups. The ROC curve was used to evaluate the CTRP9 predictive value for the OCT calcification score 3–4 group. The CTRP9 value of 89.13 pg/mL had the best balance (sensitivity 81.40%, specificity 71.60%). The AUC was 0.769 (95% CI: 0.685–0.839, *p* < 0.001, Figure 4), There was no significant difference in the results after PS matching. This confirm that the CTRP9 level decreased in patients with OCT calcification scores of 3–4 and that CTRP9 could predict calcification severity.

### 3.5. CTRP9 Predictive Value for Patient Prognosis

**Figure 5:**
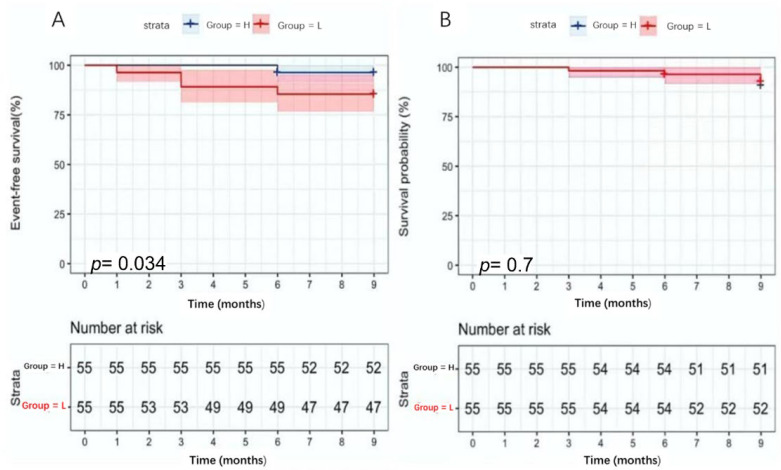
Kaplan–Meier curves analyze the event-free survival of the high-level and the low-level CTRP9 groups. According to the CTRP9 cutoff value, patients were divided into low-level and high-level CTRP9 groups. (**A**) The Kaplan–Meier curve analysis of the two groups of patients without major endpoint events; the high-level CTRP9 group had the worse outcome (*p* = 0.034). (**B**) The Kaplan–Meier curve analysis of the two groups of patients with no secondary endpoint events showed no difference between the two groups. H, high-level CTRP9 group; L, low-level CTRP9 group.

According to the CTRP9 cutoff value, 162 patients who underwent OCT were divided into the low-level (L-CTRP9) and high-level CTRP9 groups (H-CTRP9). Finally, 140 patients (86.4%) completed the 9-month follow-up. Of these, 72 patients (51.4%) were followed up in the L-CTRP9 group and 68 (48.6%) in the H-CTRP9. Major endpoint events occurred in 11 patients (15.3%) in the L-CTRP9 group and five (7.4%) in the H-CTRP9. The event-free survival rate in the H-CTRP9 group was significantly higher than that in the L-CTRP9 group (92.6% vs. 84.7%, *p* = 0.034; Figure 5). In the secondary end-events, four patients each in the L-CTRP9 (5.6%) and H-CTRP9 (5.9%) groups were readmitted for heart failure. There was no statistically significant difference between the two groups (5.6% vs. 5.9%, *p* = 0.590, Figure 5).

At 9 months, the Seattle angina and ROSE scores were assessed in both groups. The results showed that the two groups had similar physical activity limitations (66.7 vs. 71.1, *p* = 0.329), stable state of angina pectoris (75.0 vs. 100.0, *p* = 0.594), treatment satisfaction (80.0 vs. 75.0, *p* = 0.380), cognitive disease status (58.3 vs. 58.3, *p* = 0.751) and dyspnea index (2.0 vs. 1.5, *p* = 0.363) scores (Table 3). Compared with the H-CTRP9 group, angina pectoris frequency in the L-CTRP9 group increased significantly (95.0 vs. 100.0%, *p* = 0.021).

## 4. Discussion

This study demonstrated that CTRP9 expression was significantly associated with CAC, and, especially in patients with type 2 diabetes, CTRP9 played roles as a protective factor and potential predictor in CAC severity. Additionally, during the follow-up period, the incidence of major endpoint events and angina pectoris frequency was higher in the L-CTRP9 group than in the H-CTRP9 group. Thus, CTRP9 can be a valuable biomarker for CAC occurrence and severity and could predict patients’ clinical prognosis.

### 4.1. Vascular Calcification Is a Cell-Mediated Active and Highly Controllable Biological Process

Pathogenesis of vascular calcification is complex. Recent studies on vascular imaging, cell biology, and molecular biology have suggested that vascular calcification is a cell-mediated active and highly controllable biological process [26]. CAC promotes cardiovascular event occurrence and development [9]. Furthermore, Renu Virmani found that the CAC in patients with T2DM was significantly higher than that in patients without DM [26]. A study has shown that the new CAC rate in T2DM patients is significantly higher than that in patients without DM [27]. Malik et al. showed that in patients with T2DM and CAC, the 10-year atherosclerotic cardiovascular disease (ASCVD) risk was significantly higher than that in patients without CAC [28]. In summary, CAC incidence in patients with T2DM is high, with a severe degree and a rapid progression, increasing PCI difficulty and risk, and seriously affecting patient prognosis. This poses a difficult problem in coronary intervention. Determining the corresponding molecular markers has important clinical significance for identifying early calcified plaques, formulating early intervention programs, and delaying calcification progression.

### 4.2. CTRP9 Might Play an Important Role in CAC Formation in the Context of T2DM

Many studies have shown that CTRP9 can protect the cardiovascular system by regulating glucose and lipid metabolism, regulating vasodilation, inhibiting vascular inflammatory reactions, and improving endothelial dysfunction [29,30]. Previous studies have also confirmed that CTRP9 can inhibit various risk factors related to CAC in T2DM [29]. In addition, Jung et al. showed that CTRP9 was related to the degree of vascular stiffness in patients with T2DM [30], and Schurgers et al. showed [31] that in renal transplant recipients, CTRP9 levels were negatively related to the aortic calcification score (ACAI). These studies suggested that CTRP9 might play an important role in CAC formation in the context of T2DM. Therefore, this study determined the CTRP9 expression level in the control, CAC, T2DM, and T2DM with CAC groups. The results showed that plasma CTRP9 levels were significantly reduced in patients with CAC compared to those in the control group. Simultaneously, the study further showed that the plasma CTRP9 level in patients with T2DM complicated with CAC was lower than that in patients with calcification without T2DM. These results suggest that CTRP9 participates in CAC formation and may also play a more important role in CAC in the presence of glucose and lipid metabolism disorders.

Epidemiological studies show that CAC is an age- and sex-oriented disease, and the prevalence of CAC increases with age, with evidence of CAC present in more than 90% of men and 67% of women over the age of 70 [32,33]. The multivariate regression analysis results showed that age was an independent risk factor for CAC, consistent with the results of many studies [34,35]. After correcting for common clinical risk factors, plasma CTRP9 levels remained independently related to CAC, and CTRP9 is a protective factor for CAC. Therefore, this study’s results indicate that CTRP9, a protective factor, is involved in the occurrence and development of T2DM complicated by CAC. ROC curve analysis showed that plasma CTRP9 levels have good diagnostic efficacy for CAC; therefore, CTRP9 may be a potential molecular diagnostic agent for CAC. These results also suggest that the CTRP9 level is a CAC predictor and provides a theoretical basis for its clinical prevention and early intervention.

### 4.3. CTRP9 Is Associated with CAC Severity and Vascular Calcification Scores Can Pre-dict Cardiovascular Events

CAC severity is closely related to cardiovascular events and mortality. Clinically, calcification severity is usually evaluated using the CAC score. The CAC score was measured using Agatston (standard Agatston CAC score). Studies have shown that CAC scores are independently associated with ASCVD events [36]. Nixdorf et al. showed that patients with CAC scores > 400 are expected to have a 10-year incidence of ASCVD events of 13.50% [37]. In 2020, a retrospective study also found that cardiovascular and coronary heart disease mortality rates of patients with CAC scores >1000 were more than twice as high as the rate of those with CAC scores of 400–999 [38].

In terms of imaging, although coronary CT has a high sensitivity in CAC diagnosis, its plaque composition evaluation is affected by respiration and heart-rate factors. Moreover, coronary CT cannot accurately evaluate calcified lesion distribution in the lumen, increasing the interventional therapy risk. Therefore, coronary CT has certain limitations in CAC evaluation. Currently, coronary CT cannot replace invasive examinations (OCT and IVUS) concerning evaluating CAC accurately [39,40]. OCT can clearly show the coronary artery wall’s three-layer structure; therefore, it can accurately evaluate calcified lesions’ distribution. More importantly, OCT can also evaluate calcified lesions’ severity using OCT score based on quantifying the calcified plaques’ thickness, radius, and length. However, as an invasive inspection method, OCT inspection is limited due to technical and economic reasons. Therefore, it is of great clinical significance for patients with CAC to judge CAC severity using serum molecular biological markers in peripheral blood.

### 4.4. Limitations

This study has some limitations and drawbacks. As this was a single-center cross-sectional study, the patient sample size was relatively small, which may have caused bias in the results. Large-scale, multicenter follow-up studies need to be performed. The follow-up time for this study was short, therefore, it would be valuable for future studies to include, not only a larger sample size, but also a prolonged follow-up period.

## 5. Conclusions

The study results showed that major end-events in patients with high CTRP9 levels were significantly lower than those in patients with low CTRP9 levels. In addition, the Seattle angina pectoris and ROSE scores were obtained in patients with different CTRP9 levels at 9 months. The frequency of angina pectoris was significantly higher in the low-level CTRP9 group than in the high-level group. The above results show that the CTRP9 level has a good predictive value for the clinical prognosis of patients with CAC, which provides more evidence for its clinical application.

## Figures and Tables

**Table 1:** Baseline characteristics and general clinical data of each group.

Variables	Control Group	CAC Group	T2DM Group	T2DM with CAC Group	*p*
Age (years)	56.5 ± 9.1 ^b^	60.7 ± 8.4 ^a^	60.1 ± 8.2	64.4 ± 7.5 ^a,b^	<0.001
Sex (male/female)	59 (31,28)	92 (68,24)	36(26,10)	88 (60,28)	0.044
SCR (mg/dl)	67.0 (62.0,77.0)	71.0 (64.0,79.8)	78.5 (67.0,88.5) ^a^	69.0 (59.3,78.8) ^c^	0.024
Hypertension, n (%)	27 (45.8)	59 (64.1)	24 (66.7)	62 (70.5)	0.020
Smoking, n (%)	8 (13.6)	15 (16.3)	15 (41.7)	29 (33.0)	0.001
Heart failure, n (%)	2(3.4)	5 (5.4)	5 (13.9)	20 (22.7)	<0.001
Systolic BP (mmHg)	132.0 ± 18.3	137.8 ± 19.3	135.9 ± 18.5	134.6 ± 17.1	0.285
Diastolic BP (mmHg)	78.1 ± 10.7	78.5 ± 12.7	76.3 ± 10.5	77.7 ± 10.3	0.794
HR (bpm)	70.3 + 10.4 ^c^	69.0 ± 9.2^c^	65.1 ± 6.5 ^a,b^	69.2 ± 10.4 ^c^	0.010
BMI (kg/m^2^)	25.3 ± 2.9	24.6 ± 3.1^c^	26.6 ± 2.9 ^b^	25.7 ± 2.9	0.003
HGB	133.0 ± 14.7	131.3 ± 15.1	135.4 ± 13.2	131.1 ± 14.0	0.417
HbA1c (%)	5.7 (5.5,6.0)^c^	5.9 (5.5,6.1) ^c^	7.6 (6.7,8.5) ^a,b^	7.7 (6.8,8.5) ^a,b^	<0.001
GLU	4.8 (4.4,5.3)	4.7 (4.3,5.3)	6.0 (5.3,7.5) ^a,b^	6.6 (5.7,7.9) ^a,b^	<0.001
hsCRP	1.5 (0.8,5.3)	2.1 (1.0,4.9)	1.7 (1.0,4.6)	1.9 (1.1,3.5)	0.736
TC (mmol/L)	3.7 (3.3,4.4)	3.6 (3.1,4.3)	3.5 (3.1,5.0)	3.5 (2.9,4.3)	0.576
TG (mmol/L)	1.3 (1.0,1.9)	1.4 (1.0,2.0)	1.2 (1.0,1.7)	1.47 (1.1,2.2)	0.074
HDL-C (mmol/L)	1.0 (0.8,1.2)	1.0 (0.9,1.2)	1.1 (0.8,1.1)	0.9 (0.8,1.1)	0.051
LDL-C (mmol/L)	2.19 (1.8,2.7)	2.2 (1.6,2.5)	2.0 (1.7,3.0)	2.0 (1.5,2.8)	0.723
ALT	20.6 (12.8,31.5)	18.9 (12.9,25.0)	17.0 (12.6,24.8)	20.3 (13.4,33.7)	0.406
AST	19.3 (15.5,26.6)	19.5 (16.9,27.3)	15.1 (13.7,18.9) ^a,b^	18.0 (13.7,25.0) ^b^	0.001
APOA (g/L)	1.2 ± 0.2	1.2 ± 0.3	1.1 ± 0.2	1.1 ± 0.2 ^b^	0.016
ApoB (g/L)	0.8 (0.6,1.0)	0.77 (0.6,0.9)	0.70 (0.6,0.9)	0.74 (0.6,1.0)	0.573
LPa (mg/L)	11.9 (6.3,28.5)	15.4 (6.7,31.1)	11.0 (4.9,22.2)	10.3 (5.3,31.4)	0.546
MYO	45.0 (40.0,55.0)	53.5 (42.5,66.8) ^a^	48.0 (40.3,66.3)	44.0 (37.0,51.0) ^b^	0.001
CK	67.0 (50.0,89.0)	72.5 (54.0,104.8)	64.0 (42.5,89.0)	68.5 (48.0,91.0)	0.327
CKMB	18.0 (13.0,23.0)	18.0 (15.0,23.8)	17.5 (16.0,22.0)	18.0 (14.0,22.0)	0.623
LDH	173.0 (158.0,199.0)	181.5 (166.0,208.8) ^c^	166.5 (142.3,187.2) ^b^	177.0 (154.3,207.8)	0.024
GGT	23.0 (17.0,33.0)	21.5 (16.0,33.8)	20.0 (16.3,25.0)	22.5 (15.00,37.75)	0.734
β_2_MG	1.70 (1.56,2.10)	1.90 (1.60,2.20)	2.10 (1.81,2.30) ^a,b^	2.0 (1.7,2.2) ^a^	0.005
LVEF%	61.9 (59.9,66.7)	63.3 (60.5,67.3)	66.1 (60.0,70.4)	63.6 (60.1,68.0)	0.377
*E*/*A*	0.8 (0.6,1.1)	0.8 (0.6,1.1)	0.8 (0.7,1.1)	0.7 (0.6,0.9)	0.098
EDV	101.0 (84.0,115.0)	97.5 (86.0,117.5)	99.5 (81.3,121.8)	102.0 (83.2,118.8)	0.910
ESV	37.0 (29.0,44.0)	36.0 (29.3,45.0)	34.5 (26.8,46.3)	36.0 (28.0,47.8)	0.937
E/e’	11.0 (9.0,14.2)	11.5 (9.8,13.8)	12.4 (10.5,14.0)	12.9 (11.0,15.5) ^a,b^	0.039
CTRP9 (pg/mL)	206.0 (159.7,263.1) ^b,c^	80.9 (61.1,116.3) ^a^	80.7 (67.4,126.1) ^a^	68.1 (52.9,96.5) ^a,b,c^	<0.001

Note: Continuous data are presented as the mean ± SD or median (IQR). Categorical data are presented as n (%). Compared with the no coronary calcification without diabetes group, ^a^ *p* < 0.050; compared with the coronary calcification without diabetes group, ^b^ *p* < 0.050; compared with diabetes without coronary calcification group, ^c^ *p* < 0.050.

**Table 2:** Comparison of baseline characteristics between patients with CAC and patients without CAC.

Parameters	Unmatched Cohort		*p*	Matched Cohort		*p*
	No CAC (n = 95)	CAC (n = 180)		No CAC (n = 68)	CAC (n = 68)	
CTRP9 (pg/mL)	168.4 (99.3,233.4)	72.7 (56.9,102.4)	<0.001	140.0 (79.5,214.4)	71.9 (59.7,101.4)	<0.001
Age (years)	58.0 (52.0,63.0)	63.0 (57.0,68.0)	<0.001	59.2 ± 8.2	60.2 ± 8.5	0.473
HbA1c (%)	6.0 (5.6,7.1)	6.3 (5.8,7.7)	0.023	6.1 (5.6,7.4)	6.2 (5.7,7.0)	0.993
LDH	169 (151.0,192.0)	179.0 (161.0,208.0)	0.019	170 (147.200.5)	180.5 (163.2,209.0)	0.060
Male (%)	57.0 (60.0%)	128 (71.1%)	0.079	52( 76.5%)	48 (70.6%)	0.624
Hypertension, n (%)	51 (53.7%)	121 (67.2%)	0.036	48 (70.6%)	44 (64.7%)	0.632
Diabetes, n (%)	34 (35.8%)	86 (47.8%)	0.073	34 (50%)	32 (47.1%)	0.872

**Table 3:** Health status assessments at baseline and 9 months with different CTRP9 levels.

Health Status Assessments	L-CTRP9 Group (n = 72)	H-CTRP9 Group (n = 68)	*p*
SAQ Physical limitation score	66.7 (49.4,79.4)	71.1 (58.9,80.0)	0.329
SAQ Quality of life score	75.0 (50.0,100.0)	100.0 (50.0,100.0)	0.594
SAQ angina frequency score	95.0 (80.0,100.0)	100.0 (90.0,100.0)	0.021
SAQ quality of life score	80.0 (65.0,80.0)	75.0 (65.0,80.0)	0.380
SAQ Treatment satisfaction score	58.3 (41.7,75.0)	58.3 (41.7,72.9)	0.751
Rose dyspnea score	2.0 (0.0,3.0)	1.5 (0,2.0)	0.363

Note: According to the CTRP9 cutoff value, patients were divided into low-level and high-level CTRP9 groups. SAQ, Seattle Angina Questionnaire. L-CTRP9 group: low-level CTRP9; H-CTRP9, high-level CTRP9.

## Data Availability

The data used to support the findings of this study are available from the corresponding author upon request.

## Abbreviations

BMI	body mass index
HGB	hemoglobin
HbA1c	glycosylated hemoglobin A1c
GLU	fasting blood glucose
hsCRP	hypersensitive C-reactive protein
TC	total cholesterol
TG	serum triglycerides
HDL-C	high-density lipoprotein cholesterol
LDL-C	low-density lipoprotein cholesterol
SCR	creatinine
ALT	alanine transaminase
AST	aspartate transaminase
APOA	apolipoprotein A1
ApoB	apolipoprotein B
LPa	lipoprotein a
MYO	myoglobin
CK	creatine kinase
CK-MB	creatine kinase-muscle/brain
LDH	lactate dehydrogenase
GGT	γ-glutamyl transpeptidase
β2MG	β2 microglobulin
LVEF	left ventricular ejection fraction
EDV	end-diastolic volume
ESV	end-systolic volume
CTRP9	C1q/TNF-related protein 9
